# Supplementary material for: Quantitative structured illumination microscopy via a physical model-based background filtering algorithm reveals actin dynamics
Source: Nat Commun. 2023 May 29;14:3089. doi: 10.1038/s41467-023-38808-8 (PMC10227022; doi:10.1038/s41467-023-38808-8)
Supplement: Supplementary file 1 — Supplementary Information [file 41467_2023_38808_MOESM1_ESM.pdf]

Supplementary Information

Quantitative structured illumination microscopy via a physical model-based background filtering algorithm reveals actin dynamics

Yanquan Mo[1], Kunhao Wang[2], Liuju Li[1], Shijia Xing[1], Shouhua Ye[3], Jiayuan Wen[3], Xinxin Duan[4], Ziying Luo[3], Wen Gou[5], Tongsheng Chen[2], Yu-Hui Zhang[4], Changliang Guo[1], Junchao Fan[5]✉ & Liangyi Chen[1,6,7,8]✉

[1]State Key Laboratory of Membrane Biology, Center for Life Sciences, College of Future Technology, Peking University, Beijing 100871, China.

[2]Key Laboratory of Laser Life Science, Ministry of Education, College of Biophotonics, South China Normal University, Guangzhou 510631, China.

[3]Guangzhou Computational Super-resolution Biotech Co.,Ltd, Guangzhou 510535, China.

[4]Britton Chance Center and MOE Key Laboratory for Biomedical Photonics, Wuhan National Laboratory for Optoelectronics, Huazhong University of Science and Technology, Wuhan 430074, China.

[5]Chongqing Key Laboratory of Image Cognition, College of Computer Science and Technology, Chongqing University of Posts and Telecommunications, Chongqing 400065, China.

[6]PKU-IDG/McGovern Institute for Brain Research, Beijing 100871, China.

[7]Beijing Academy of Artificial Intelligence, Beijing 100871, China.

[8]National Biomedical Imaging Center, Beijing 100871, China.

✉Correspondence to: fanjc@cqupt.edu.cn, and lychen@pku.edu.cn

Supplementary Note 1

Principle of BF-SIM. The out-of-focus fluorescence signals are diffused to the imaging focal plane and then accepted by the camera (Fig. 1a), which can be described by the three-dimensional point spread function (3D PSF). Therefore, the imaging model of the SIM with the imaging background can be written as:

$$d(\mathbf{r}) = [g(\mathbf{r}, z_{in}) \cdot I(\mathbf{r}, z_{in})] \otimes h(\mathbf{r}, z_{in}) + [g(\mathbf{r}, z_{out}) \cdot I(\mathbf{r}, z_{out})] \otimes h(\mathbf{r}, z_{out}), \quad (1)$$

where $d(\mathbf{r})$ is the emission distribution detected by the camera; $g(\mathbf{r}, z_{in})$, $I(\mathbf{r}, z_{in})$ and $h(\mathbf{r}, z_{in})$ are the actual spatial distribution of the object labeled with fluorophores, the sinusoidal intensity pattern for illumination, and the PSF within the range in-focus z_{in}, respectively; and in the same way, $g(\mathbf{r}, z_{out})$, $I(\mathbf{r}, z_{out})$ and $h(\mathbf{r}, z_{out})$ are the actual objects labeled with fluorophores, the sinusoidal illumination pattern, and the PSF with the range out-of-focus z_{out}, respectively. $\mathbf{r} = (x, y)$ is the 2D space position vector, the *z*-axis is the optical axis, and $\otimes$ is the convolution operator.

Firstly, we assumed $I(\mathbf{r}, z_{in})$ was the same as $I(\mathbf{r}, z_{out})$ in 2D-SIM. For 2D-SIM, it uses two coherent beams with the same polarization direction (such as S-polarized light) for illumination, as shown in Supplementary Fig. 1. Assuming that the wavevectors of the two beams are $\mathbf{k_1}$ and $\mathbf{k_2}$, respectively, the two beams can be expressed as

$$\mathbf{E_1} = E_1 \exp[\mathrm{i}(\mathbf{k_1} \cdot \mathbf{R} - \omega t)], \quad (2)$$
$$\mathbf{E_2} = E_2 \exp[\mathrm{i}(\mathbf{k_2} \cdot \mathbf{R} - \omega t + \varphi)],$$

where E_1 and E_2 are the amplitudes of two coherent beams, ω is the angular frequency, and φ is the phase difference between the two beams that produces the illumination patterns. $\mathbf{R} = (x, y, z)$ is the space position vector. Thus, the illumination intensity I could be written as:

$$I = |\mathbf{E_1} + \mathbf{E_2}|^2 = |\mathbf{E_1}|^2 + |\mathbf{E_2}|^2 + 2|\mathbf{E_1} \cdot \mathbf{E_2}| = I_1 + I_2 + 2\sqrt{I_1 I_2}\cos[(\mathbf{k_1} - \mathbf{k_1}) \cdot \mathbf{R} + \varphi], \quad (3)$$

where I_1 and I_2 are the illumination intensities of two beams. According to the properties of coherent light $|\mathbf{k_1}|$=$|\mathbf{k_2}| = k_0$, $I_1 = I_2 = I_0$. Therefore, the illumination intensity after interference could be represented as follows:

$$I = 2I_0 + 2I_0\cos[2k_0\sin\theta \cdot x + 2k_0\sin\theta \cdot y + \varphi]. \quad (4)$$

As seen from the above equation, the illumination intensity distribution is independent of the *z*-axis; in other words, the illumination at different depths of the *z*-axis of 2D-SIM is the same when we do not consider the illumination aberration. Therefore, under the 2D-SIM illumination, the BF-SIM model is consistent with the actual situation. To facilitate calculation, the illumination pattern is rewritten as:

$$I(\mathbf{r}) = 1 + c \cdot \cos(2\pi p \cdot \mathbf{r} + \varphi), \quad (5)$$

where c is the modulation depth, p is the pattern period, and φ is the initial phase. The illumination of 2D

SIM is commonly composed of 9 sinusoidal patterns in 3 orientations and 3 phases, and the phase difference between these 3 initial phases is 2π/3. Therefore, Model (1) is rewritten as

$$d(\mathbf{r}) = [g(\mathbf{r}, z_{in}) \cdot I(\mathbf{r})] \otimes h(\mathbf{r}, z_{in}) + [g(\mathbf{r}, z_{out}) \cdot I(\mathbf{r})] \otimes h(\mathbf{r}, z_{out}). \quad (6)$$

And in the frequency domain:

$$\begin{aligned} D(\mathbf{k}) = {} & G_{in}(\mathbf{k})H_{in}(\mathbf{k}) + \frac{c}{2}G_{in}(\mathbf{k}-p)H_{in}(\mathbf{k})e^{-i\varphi} + \frac{c}{2}G_{in}(\mathbf{k}+p)H_{in}(\mathbf{k})e^{i\varphi} \\ & + G_{out}(\mathbf{k})H_{out}(\mathbf{k}) + \frac{c}{2}G_{out}(\mathbf{k}-p)H_{out}(\mathbf{k})e^{-i\varphi} + \frac{c}{2}G_{out}(\mathbf{k}+p)H_{out}(\mathbf{k})e^{i\varphi}, \end{aligned} \quad (7)$$

where $D(\mathbf{k})$, $G_{in}(\mathbf{k})$, $H_{in}(\mathbf{k})$, $G_{out}(\mathbf{k})$, and $H_{out}(\mathbf{k})$ are the spectra of $d(\mathbf{r})$, $g(\mathbf{r}, z_{in})$, $h(\mathbf{r}, z_{in})$, $g(\mathbf{r}, z_{out})$ and $h(\mathbf{r}, z_{out})$, respectively. The variable $\mathbf{k}$ is the coordinate in the frequency domain. For an illumination orientation of SIM, three raw images with different initial phases were taken and denoted as D_1, D_2, and D_3. The step of the high- and low-frequency spectrum components separation in the Wiener reconstruction is as follows:

$$\begin{bmatrix} S_0(\mathbf{k}) \\ S_{-1}(\mathbf{k}) \\ S_{+1}(\mathbf{k}) \end{bmatrix} = \begin{bmatrix} 1 & e^{-0i} & e^{0i} \\ 1 & e^{-\frac{2\pi}{3}i} & e^{\frac{2\pi}{3}i} \\ 1 & e^{-\frac{4\pi}{3}i} & e^{\frac{4\pi}{3}i} \end{bmatrix}^{-1} \begin{bmatrix} D_1(\mathbf{k}) \\ D_2(\mathbf{k}) \\ D_3(\mathbf{k}) \end{bmatrix}$$
$$= \begin{bmatrix} H_{in}(\mathbf{k}) \cdot G_{in}(\mathbf{k}) \\ H_{in}(\mathbf{k}) \cdot G_{in}(\mathbf{k}-p)(\frac{c_1}{2}e^{-i\varphi}) \\ H_{in}(\mathbf{k}) \cdot G_{in}(\mathbf{k}+p)(\frac{c_1}{2}e^{i\varphi}) \end{bmatrix} + \begin{bmatrix} H_{out}(\mathbf{k}) \cdot G_{out}(\mathbf{k}) \\ H_{out}(\mathbf{k}) \cdot G_{out}(\mathbf{k}-p)(\frac{c_1}{2}e^{-i\varphi}) \\ H_{out}(\mathbf{k}) \cdot G_{out}(\mathbf{k}+p)(\frac{c_1}{2}e^{i\varphi}) \end{bmatrix}. \quad (8)$$

The high-order spectral components $S_{-1}(\mathbf{k})$ and $S_{+1}(\mathbf{k})$ not only contain focus fluorescence signals but are also affected by the background originating from the out-of-focus fluorescence, which is the source of high-frequency bright patchy features in the SR image spectrum after the frequency shift.

In Eq. (6), because $h(\mathbf{r}, z_{out})$ represents a highly low-frequency part of the background, we can assume:

$$[g(\mathbf{r}, z_{out}) \cdot I(\mathbf{r})] \otimes h(\mathbf{r}, z_{out}) \approx [g(\mathbf{r}, z_{in}) \cdot I(\mathbf{r})] \otimes h(\mathbf{r}, z_{out}). \quad (9)$$

Therefore, samples do not need to have the same distribution at different depths but would have approximately the same distribution after the convolution with $h(\mathbf{r}, z_{out})$. We used multilayer 2D-SIM to image 3D cell samples with different distributions at different depths. Using ±400 nm in the middle layer as in-focus signals and other layers as the out-of-focus background[2], we calculated the similarity between $[g(\mathbf{r}, z_{in}) \cdot I(\mathbf{r})] \otimes h(\mathbf{r}, z_{out})$ and $[g(\mathbf{r}, z_{out}) \cdot I(\mathbf{r})] \otimes h(\mathbf{r}, z_{out})$ (Supplementary Fig. 2). As can be seen from the statistical results, the hypothesis $[g(\mathbf{r}, z_{in}) \cdot I(\mathbf{r})] \otimes h(\mathbf{r}, z_{out}) \approx [g(\mathbf{r}, z_{out}) \cdot I(\mathbf{r})] \otimes h(\mathbf{r}, z_{out})$ is approximately satisfied.

Finally, we tested whether it was possible to estimate out-of-focal fluorescence intensity with the image from a single 2D layer. Thus, we denoted a single-layer 2D-SIM image as $g(\mathbf{r}, z_{focus})$, and

calculated $[g(\mathbf{r}, z_{focus}) \cdot I(\mathbf{r})] \otimes h(\mathbf{r}, z_{out})$. As shown in Supplementary Fig. 2c-2f, $[g(\mathbf{r}, z_{focus}) \cdot I(\mathbf{r})] \otimes h(\mathbf{r}, z_{out})$ was almost the same as $[g(\mathbf{r}, z_{in}) \cdot I(\mathbf{r})] \otimes h(\mathbf{r}, z_{out})$. Thus, we have the following approximate relationship:

$$[g(\mathbf{r}, z_{focus}) \cdot I(\mathbf{r})] \otimes h(\mathbf{r}, z_{out}) \approx [g(\mathbf{r}, z_{in}) \cdot I(\mathbf{r})] \otimes h(\mathbf{r}, z_{out}) \approx [g(\mathbf{r}, z_{out}) \cdot I(\mathbf{r})] \otimes h(\mathbf{r}, z_{out}). \quad (10)$$

Therefore, the final imaging model of Eq.(1) can be rewritten as follows:

$$d(\mathbf{r}) = [g(\mathbf{r}, z_{focus}) \cdot I(\mathbf{r})] \otimes h(\mathbf{r}, z_{in}) + [g(\mathbf{r}, z_{focus}) \cdot I(\mathbf{r})] \otimes h(\mathbf{r}, z_{out}). \quad (11)$$

Then, we may estimate the out-focal fluorescence from the in-focal single-plane image.

Hence, the in-focal signal distribution is equal to the raw image minus the defocused background:

$$g(\mathbf{r}, z_{out}) \cdot I(\mathbf{r}) = iFt\left\{\frac{Ft\{d(\mathbf{r})\}}{Ft\{h(\mathbf{r}, z_{in})\} + Ft\{h(\mathbf{r}, z_{out})\}}\right\} = iFt\left\{\frac{D(\mathbf{k})}{H_{in}(\mathbf{k}) + H_{out}(\mathbf{k})}\right\}, \quad (12)$$

$$d_{in}(\mathbf{r}) = d(\mathbf{r}) - [g(\mathbf{r}, z_{out}) \cdot I(\mathbf{r})] \otimes h(\mathbf{r}, z_{out}) = d(\mathbf{r}) - iFt\left\{D(\mathbf{k}) \cdot \frac{H_{out}(\mathbf{k})}{H_{in}(\mathbf{k}) + H_{out}(\mathbf{k})}\right\}, \quad (13)$$

where Ft and iFt are Fourier transform and the inverse Fourier transform.

Finally, we can use the $d_{in}(\mathbf{r})$, which dislodges out-of-focus fluorescence, for subsequent SR reconstruction. However, because it is challenging to obtain the actual corresponding $H_{out}(\mathbf{k})$ and $H_{in}(\mathbf{k})$, we use the simulated 3D PSF built from the PSF Generator plugin in ImageJ[1]. According to the Rayleigh criterion ($\Delta_{\text{Rayleigh},z} = 2n\lambda/NA^2$)[3], under the same microscope imaging parameters (the wavelength of illumination laser is 488-640 nm, and the effective NA of the objective lens corresponding to 2D-SIM is 1.4-1.5)[4], the z-axis resolution of an ordinary 3D microscope is approximately 800 nm. Therefore, fluorescence signals within ± 0.4 μm the axial distance from the focal plane are regarded as in-focused, while those outsides of ± 0.4 μm are considered to be out-of-focus signals. Meanwhile, the total intensity of PSF corresponding to the depth of $z = 4$ μm is approximately 1% of that at $z = 0$ μm, thus the fluorescence in the range -0.4--4 μm and 0.4-4 μm are selected as the background, while the much deeper fluorescence can be ignored. Similarly, $H_{in}(\mathbf{k})$ and $H_{out}(\mathbf{k})$ also use the same depth to calculate. And for specific calculation, $H_{in}(\mathbf{k})$ and $H_{out}(\mathbf{k})$ are the sum by stacking the 2D OTF of each layer corresponding to the 2D PSF.

In addition, we have examined the effects of $H_{in}(\mathbf{k})$, $H_{out}(\mathbf{k})$, $H_{out}(\mathbf{k})/(H_{in}(\mathbf{k}) + H_{out}(\mathbf{k}))$, background decomposition and the final reconstructed SR images when the focal range was set at ±0.2 μm, ±0.4 μm, ±0.6 μm, ±0.8 μm, while the depth of the total PSF did not change (Supplementary Fig. 3). From this experiment, we confirmed that ±0.4 μm was also a robust choice, which was suitable for most organelle

data and had good background removal effect without losing weak signals.

Thus the implementation procedure of our BF-SIM algorithm is summarized below.

Algorithm: BF-SIM

Step 1: Set $T_{in} = 0.4$ μm, $T_{out} = 4$ μm

Step 2: Generate 3D-PSF $h(\mathbf{r}, z)$

Implement 2D Fourier transform: $H(\mathbf{k}, z_i) = Ft(h(\mathbf{r}, z_i))$

Step 3: Compute

$$H_{in}(\mathbf{k}) = \sum_{z_i=-T_{in}}^{z_i=T_{in}} H(\mathbf{k}, z_i),$$

$$H_{out}(\mathbf{k}) = \sum_{z_i=-T_{out}}^{z_i=-T_{in}} H(\mathbf{k}, z_i) + \sum_{z_i=T_{in}}^{z_i=T_{out}} H(\mathbf{k}, z_i)$$

Step 4: Obtain $d_{in}(\mathbf{r})$ using Eq. (12), (13)

Step 5: Take $d_{in}(\mathbf{r})$ as the raw image and then use an arbitrary method, such as Wiener-SIM, to reconstruct.

We emphasized that our BF background suppression model was a compromise method for the absence of axial sample distribution under the 2D-SIM conditions. Thus, BF-SIM deals with 2D SIM datasets in a plane-by-plane manner. Under the 3D-SIM with three-beam illumination, the illumination on the z-axis has different intensities at different depths, which does not meet our assumptions. In addition, 3D-SIM uses 3D PSF to perform 3D deconvolution of images during reconstruction, which essentially redistributes the background to its focal plane. Therefore, we do not recommend BF-SIM for background suppression in 3D-SIM. Nevertheless, the BF method was also used to process images without much fluorescence background (Supplementary Fig. 5). Thus, we suggest that users may prudently apply the BF method to image datasets of other microscopic modalities.

Quantification of SR image contrasts. Different algorithms generate SR reconstructions with different intensities, which may have different baselines. To better compare the differences between weak signals, we normalize the signal intensity distribution v:

$$v'(i) = \frac{v(i) - \min(v)}{\max(v) - \min(v)} \tag{14}$$

where $\max(v)$ and $\min(v)$ are the maximum and minimum values of the intensity distribution, respectively, i is the coordinate, and v' is the normalized signal intensity. The raw signal is divided by the maximum signal intensity for normalization to the maximum signal.

Then, we use the image contrast ratio to evaluate background suppression in strong or weak signals:

$$\mathrm{CR} = \sum_{\delta} \delta(m,n)^2 P_{\delta}(m,n)\,, \tag{15}$$

where $\delta(m,n) = |m-n|$ is the difference between the gray values of adjacent pixels, m and n are the image gray values of adjacent pixels, and P_{δ} is the distribution probability of pixels whose gray value difference between adjacent pixels is δ. The size of adjacent areas is set to four neighbors. However, the gray values of the results obtained by different reconstruction methods are also different. To compare the contrast ratio differences between these methods, we first normalized the maximum gray values of each SR image to 1 before calculating the contrast ratios using Eq.15. We also normalized the maximum value of the contrast ratios to 1 after contrast ratio calculations to better display their differences.

Supplementary Figures

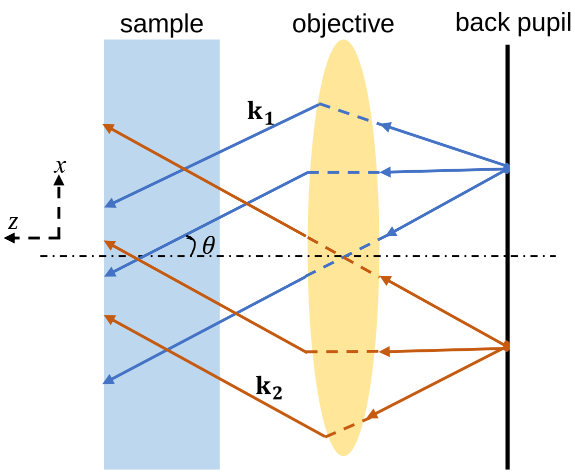

Supplementary Fig. 1 | The schematic illustration of two-beam illumination for 2D-SIM.

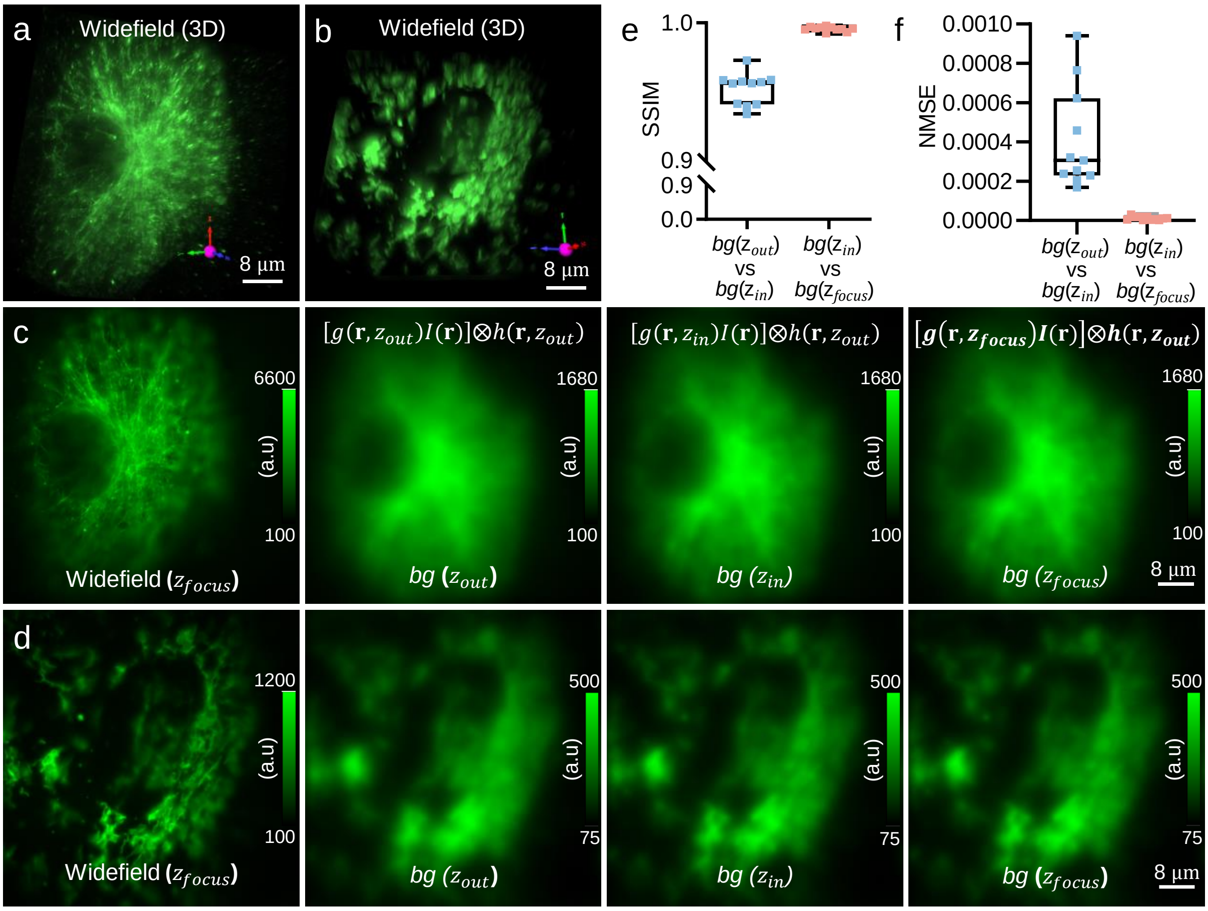

Supplementary Fig. 2 | In-focal widefield distributions of 3D samples and the defocus background calculated from different image depths. a, **b**, 3D distributions of tubulin (β-tubulin E7) and mitochondria (PK Mito Deep Red) were acquired by multilayer 2D-SIM (55 layers, at an axial interval of 100 nm) but reconstructed with the widefield method. **c**, **d**, Widefield images of the middle layer as $z=z_{focus}$. We calculated $[g(\mathbf{r},z_{out})\cdot I(\mathbf{r})]\otimes h(\mathbf{r},z_{out})$, $[g(\mathbf{r},z_{in})\cdot I(\mathbf{r})]\otimes h(\mathbf{r},z_{out})$, and $[g(\mathbf{r},z_{focus})\cdot I(\mathbf{r})]\otimes h(\mathbf{r},z_{out})$ to be different backgrounds as $bg(z_{out})$, $bg(z_{in})$ and $bg(z_{focus})$. All data were set to z_{out}=4 µm. **e**, **f**, The structural similarity coefficients (SSIMs) and the normalized root mean square errors (NMSEs) between different backgrounds ($bg(z_{out})$ versus $bg(z_{in})$, SSIM: 0.952±0.003, NMSE: 0.00041±0.00007; $bg(z_{in})$ versus $bg(z_{focus})$, SSIM: 0.996±0.001, NMSE: 0.0000±0.000001 (n=11 biologically independent cells).

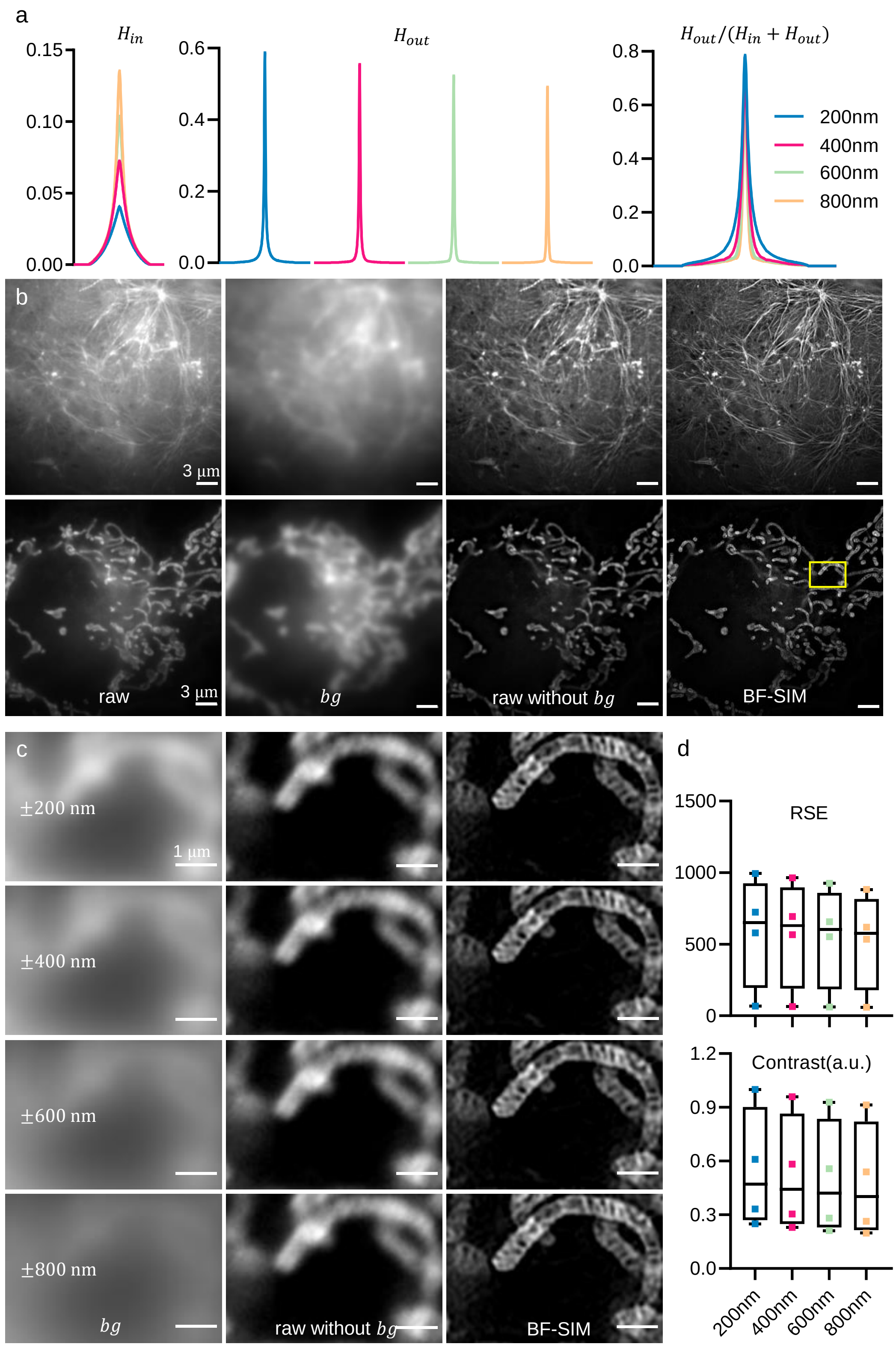

Supplementary Fig. 3 | Different H_{in}, H_{out} and $H_{out}/(H_{in}+H_{out})$ corresponding to different in-focus depths of BF-SIM. a, The H_{in}, H_{out} and $H_{out}/(H_{in}+H_{out})$ distributions when the in-focus depth z_{in} =±0.2 μm, ±0.4 μm, ±0.6 μm, ±0.8 μm, respectively. **b**, The separation results of the out-of-focus background, the in-focus signals, and final BF-SIM SR images of actin (COS-7 cell labeled with LifeAct-EGFP) and mitochondria (COS-7 cell labeled with MitoTracker Green) at z_{in} =± 0.4 μm. **c**, The enlarged region of mitochondria in (**b**), the out-of-focus background, the raw image without background, and the final BF-SIM results when z_{in} =±0.2 μm, ±0.4 μm, ±0.6 μm, ±0.8 μm respectively. **d**, RSEs of SR images with different in-focal ranges were calculated using widefield as the reference, while image contrasts were shown below (n=4 biologically independent cells).

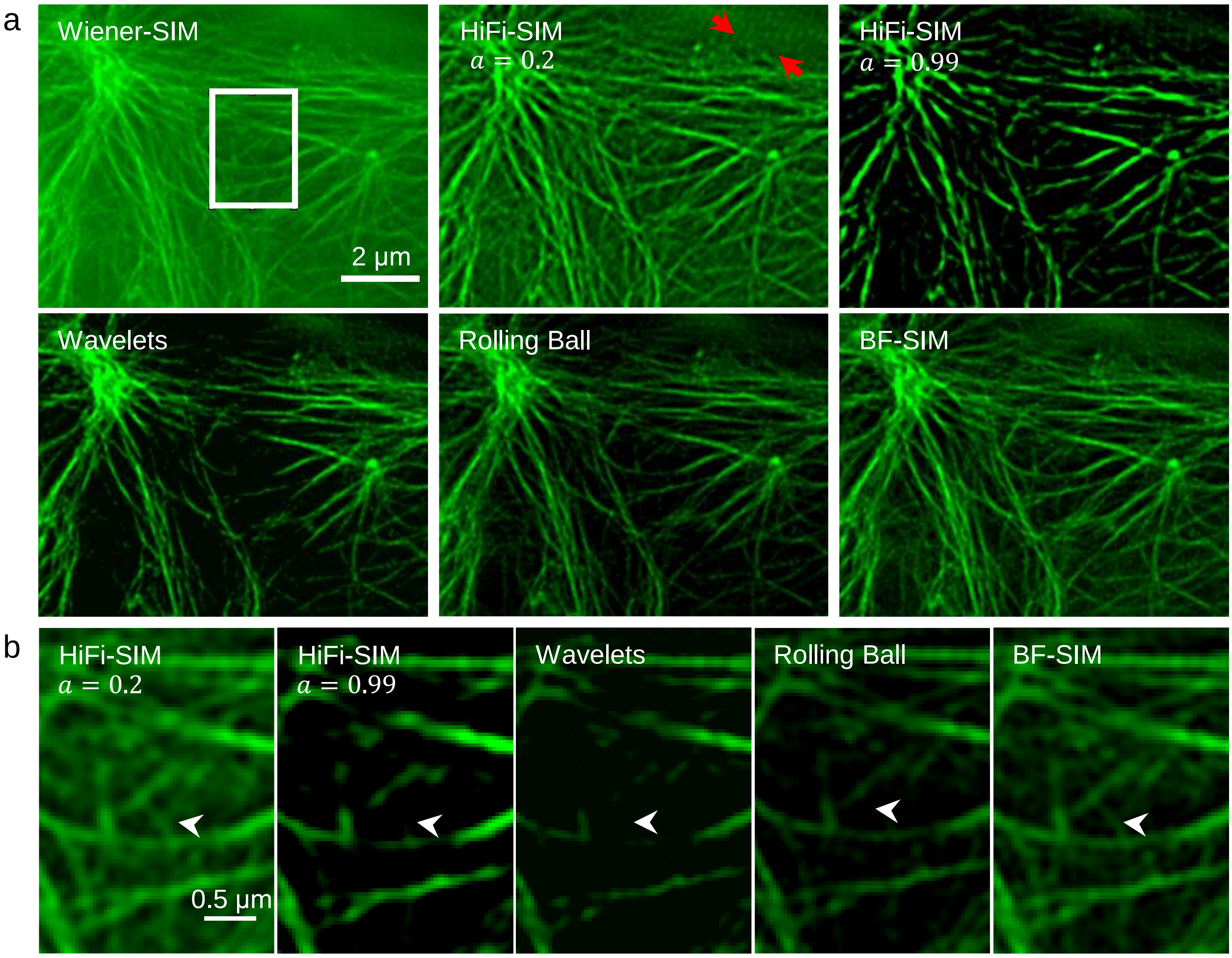

Supplementary Fig. 4 | Comparison of the effects of different methods in removing excessive background before SIM reconstruction. a, Actin images from the upper-left ROI in Fig. 1 were processed with different background suppression methods. HiFi-SIM with an oversmall parameter (attStrength=0.2) failed to remove the background effectively, while some faint stripe artifacts persisted (red arrows); at a larger value of attStrength (0.99), the background suppression was overdone and again led to the removal of weak signals (upper panels). Before the subsequent Wiener reconstruction, we also removed the background from the raw images with standard methods, including wavelets and the rollingball. Both methods removed weak signals (arrowheads, bottom panels). **b,** Enlarged ROI in the white box in **(a)**.

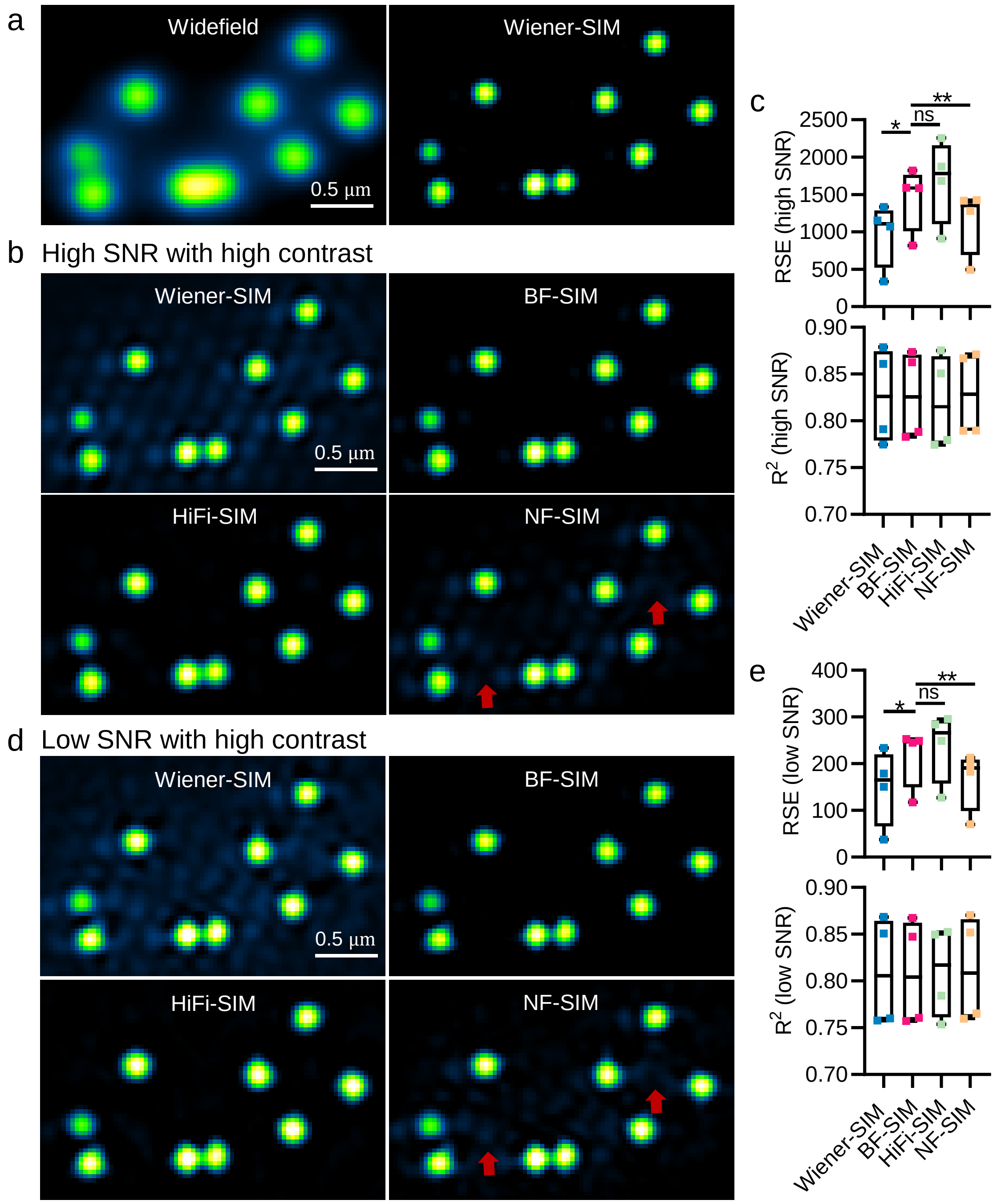

Supplementary Fig. 5 | Fluorescence beads of various SNRs reconstructed with different background suppression methods. a, Fluorescence beads with diameters of 200 nm observed under the widefield (left) and Wiener-SIM (right). **b**, **c**, Different SR reconstructions of beads with 10 ms exposures. We calculated RSEs, and the correlation coefficients (R^2s) between increases in the fluorescence intensities under SIM (ΔF_{SIM}) and those under the widefield ($\Delta F_{widefield}$). The RSEs and R^2s were: Wiener-SIM (973.10±218.82, 0.826±0.026), BF-SIM (1453.13±218.28, 0.827±0.024), HiFi-SIM (1679.83±282.95, 0.820±0.025), NF-SIM (1153.83±222.86, 0.829±0.023), n=4 biologically independent samples. **d**, **e**, Different SR reconstructions of beads with 1 ms exposures. The RSEs and R^2s were: Wiener-SIM (150.01±41.36, 0.809±0.029), BF-SIM (216.05±32.93, 0.808±0.029), HiFi-SIM (238.96±38.59, 0.810±0.025), NF-SIM (166.03±32.47, 0.812±0.029), n=4 biologically independent samples. Images in (**b, d**) were displayed with the same contrast as BF-SIM. In this experiment, the parameter of NF-SIM is attStrength=0.999, attwidth=2. We used the two-tailed paired Student's *t*-test for data in **(c, e)**, and no adjustments were made for multiple comparisons. $*p < 0.05$, $**p < 0.01$. p=0.0174 (BF-SIM vs Wiener-SIM) and p=0.0085 (BF-SIM vs NF-SIM) in (**c**), p=0.0278 (BF-SIM vs Wiener-SIM) and p=0.0017 (BF-SIM vs NF-SIM) in (**e**).

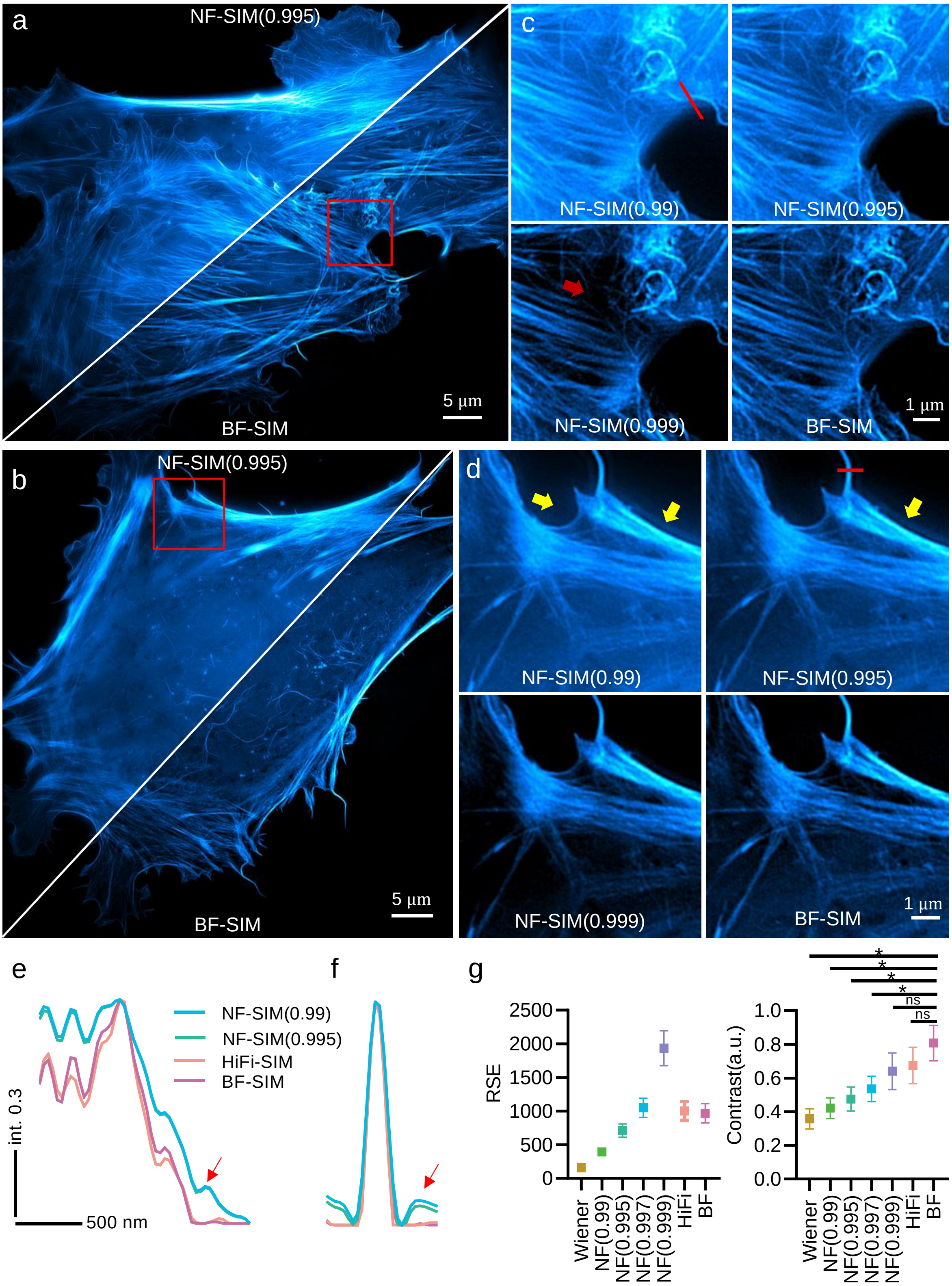

Supplementary Fig. 6 | Back-to-back comparisons among BF-SIM, HiFi-SIM, and NF-SIM. a, b, The actin filaments (LifeAct-EGFP) of a living U2OS cell under BF-SIM and NF-SIM with the best attsStrength parameter of 0.995. **c**, **d**, The red boxes in (**a**, **b**) were enlarged and reconstructed with BF-SIM and NF-SIM with different parameters. **e**, **f**, Normalized intensity profiles along the red lines in (**c**, **d**). **g**, RSEs, and contrasts of actin under different reconstructions (n=3 biologically independent cells). *, $p < 0.05$, according to the two-tailed paired student *t*-test. p=0.0104 (BF vs Wiener), p=0.0147 (BF vs NF 0.99), p=0.0152 (BF vs NF 0.995), p=0.0209 (BF vs NF 0.997). The number in brackets after NF-SIM (NF) indicated the value of the parameter attsStrength.

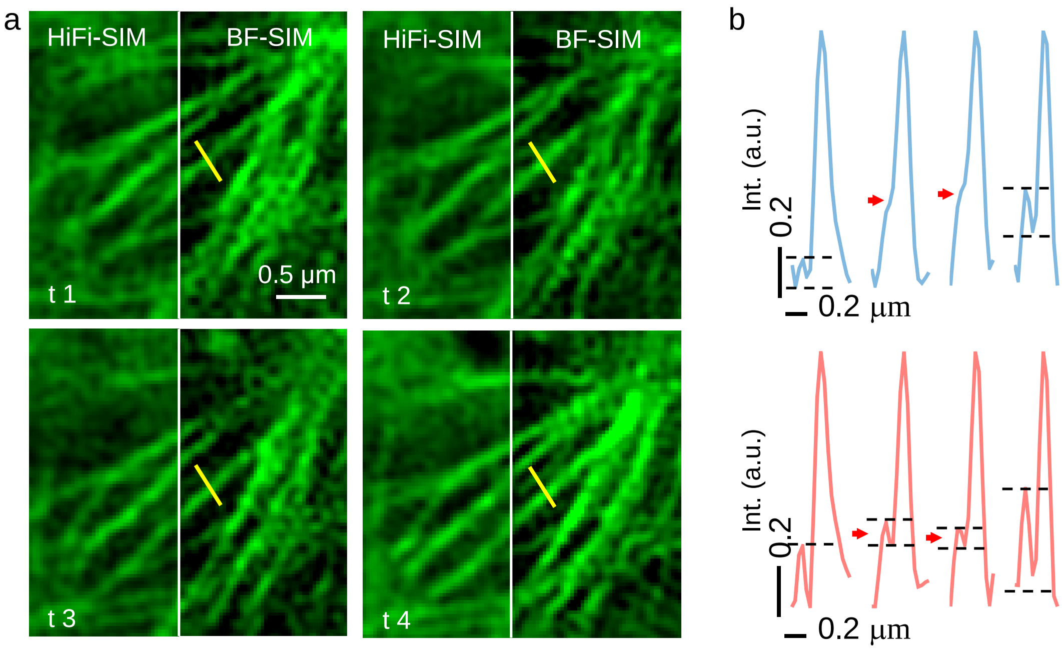

Supplementary Fig. 7 | BF-SIM suppresses the background and retains weak signals during reconstruction. a, Four consecutive time points (t1, t2, t3, and t4) of the yellow boxed ROI in Fig. 1 were enlarged, and HiFi-SIM and BF-SIM SR images are shown. **b,** The upper panel shows the normalized intensities along the yellow line in the HiFi-SIM, while the bottom panel shows those under the BF-SIM. BF-SIM resolved the weak signal at all time points with better contrast (red arrows).

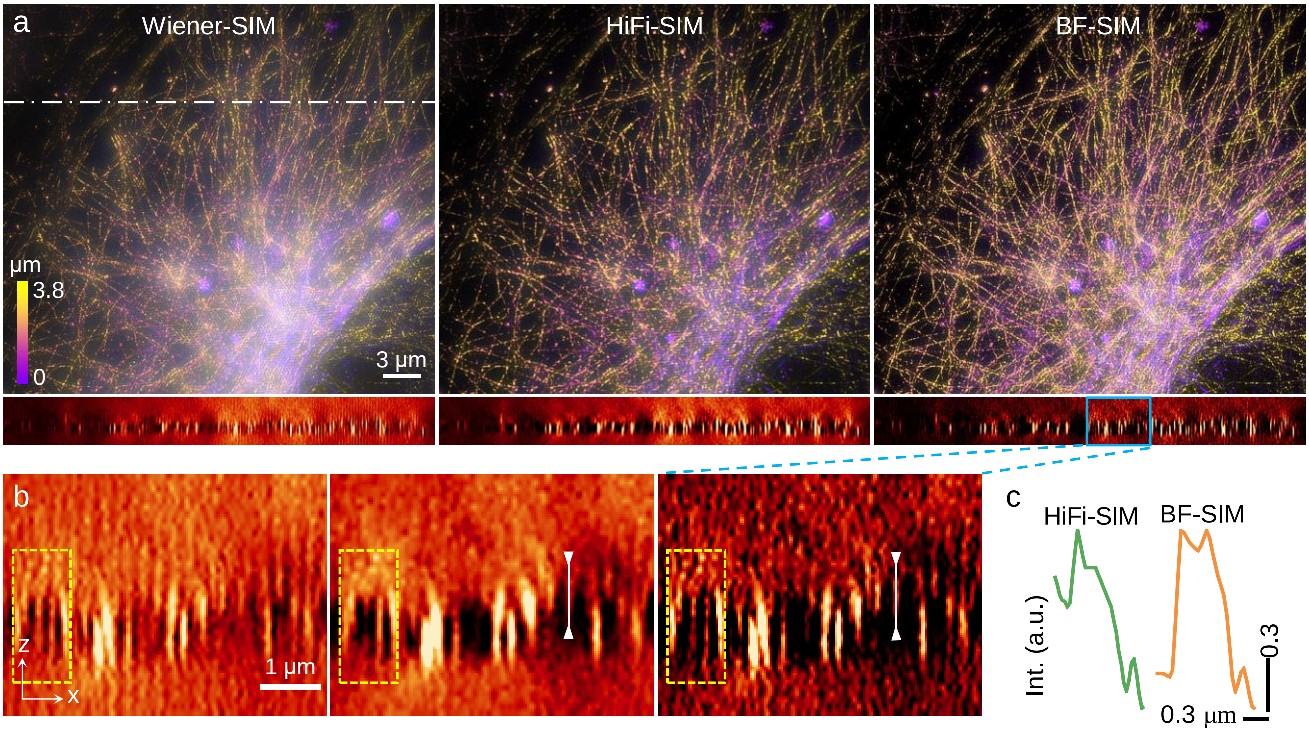

Supplementary Fig. 8 | BF-SIM improves the optical section capability of multilayer two-beam interference SIM. A fixed BSC-1C cell was immunostained with β-tubulin E7 and imaged plane-by-plane under two-beam interference SIM for 3.8 μm (at an axial interval of 100 nm). **a,** The x-y and x-z (at the white dotted line) views of the SR volume were reconstructed plane-by-plane by three methods. **b,** Enlarged x-z orthogonal view in **(a)**. In the yellow dashed box, one intricate structure along the z-axis disappeared in the HiFi-SIM reconstruction but remained in the Wiener-SIM and BF-SIM reconstructions. **c,** Normalized intensity profiles along the white line in **(b)**.

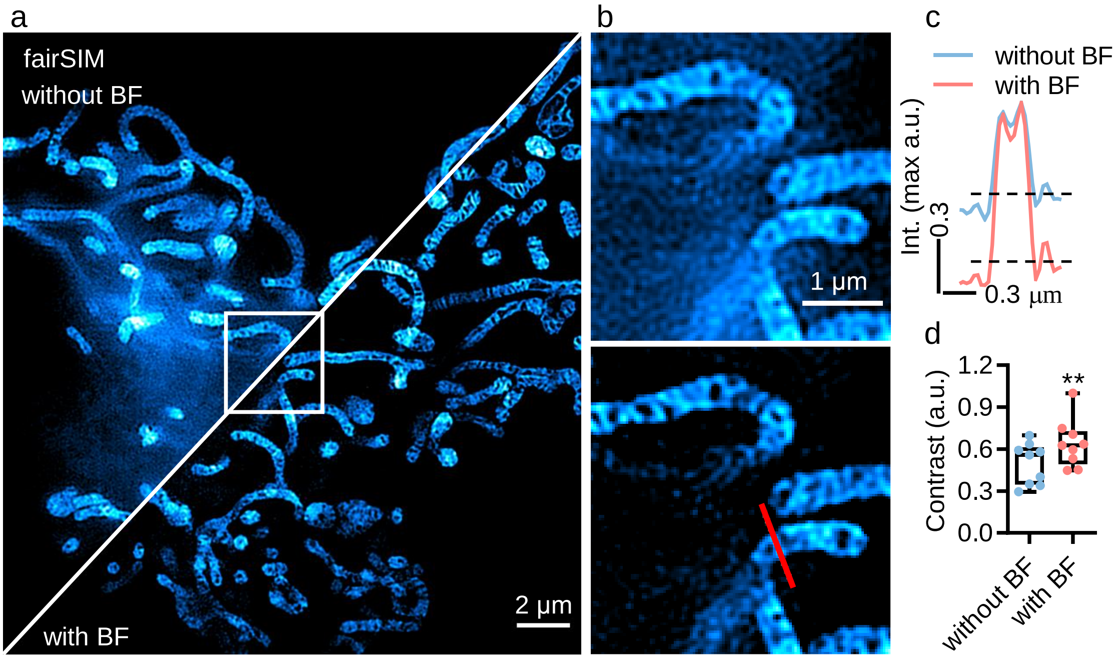

Supplementary Fig. 9 | BF enables better fairSIM reconstructions. a, Adding BF to fairSIM significantly suppressed the background and hammer-stroke artifacts in SR reconstructions. The mitochondria of living COS-7 cells were labeled with MitoTracker Green. **b,** Enlarged ROI in the white box in **(a)**. The upper and bottom panels without or with BF preprocessing, respectively. **c,** Maximum normalized intensity profiles along the red line in **(b)**. **d,** Normalized contrast ratios of mitochondrial images without (blue) and with (red) BF preprocessing (n=3 biologically independent cells × 3 frames). We used the two-tailed paired Student's *t*-test for the data in **(d)**. $** \ p < 0.01$ and p=0.0032.

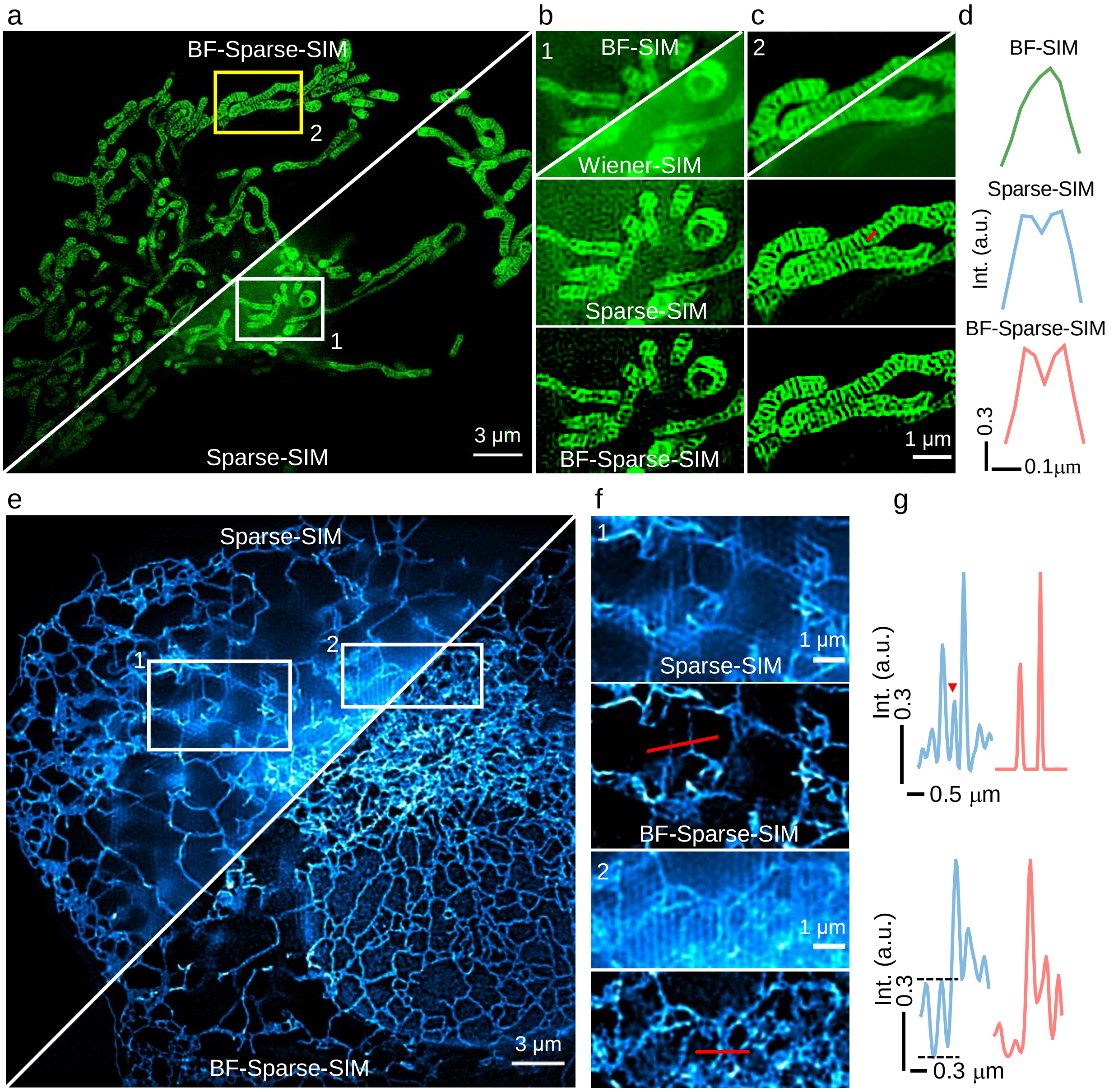

Supplementary Fig. 10 | Adding the BF step to Sparse-SIM improves contrast and suppresses fixed pattern artifacts. a, The mitochondria of a living COS-7-cell labeled by MitoTracker Green were observed under Sparse-SIM without (upper-left) or with (bottom right) BF preprocessing. **b,c,** Enlarged ROIs in **(a)** show regions of both densely and sparsely populated mitochondria. **d,** Normalized intensity profiles along the red line in **(c)**. Sparse-SIM could distinguish between two closely connected mitochondrial inner ridges, and the signal background contrast was higher with background suppression. **e,** The endoplasmic reticulum labeled with mCherry-Cytb5ER in living COS-7 cells was observed under Sparse-SIM without (upper-left) or with (bottom right) BF preprocessing. **f,** Enlarged ROIs in the white boxes in **(e)**. **g** Normalized intensity profiles along the red line in **(f)**. The red arrowhead and dotted lines indicated periodic artifacts caused by the defocus background and enhanced by the sparse deconvolution. These artifacts were eliminated by the BF preprocessing step.

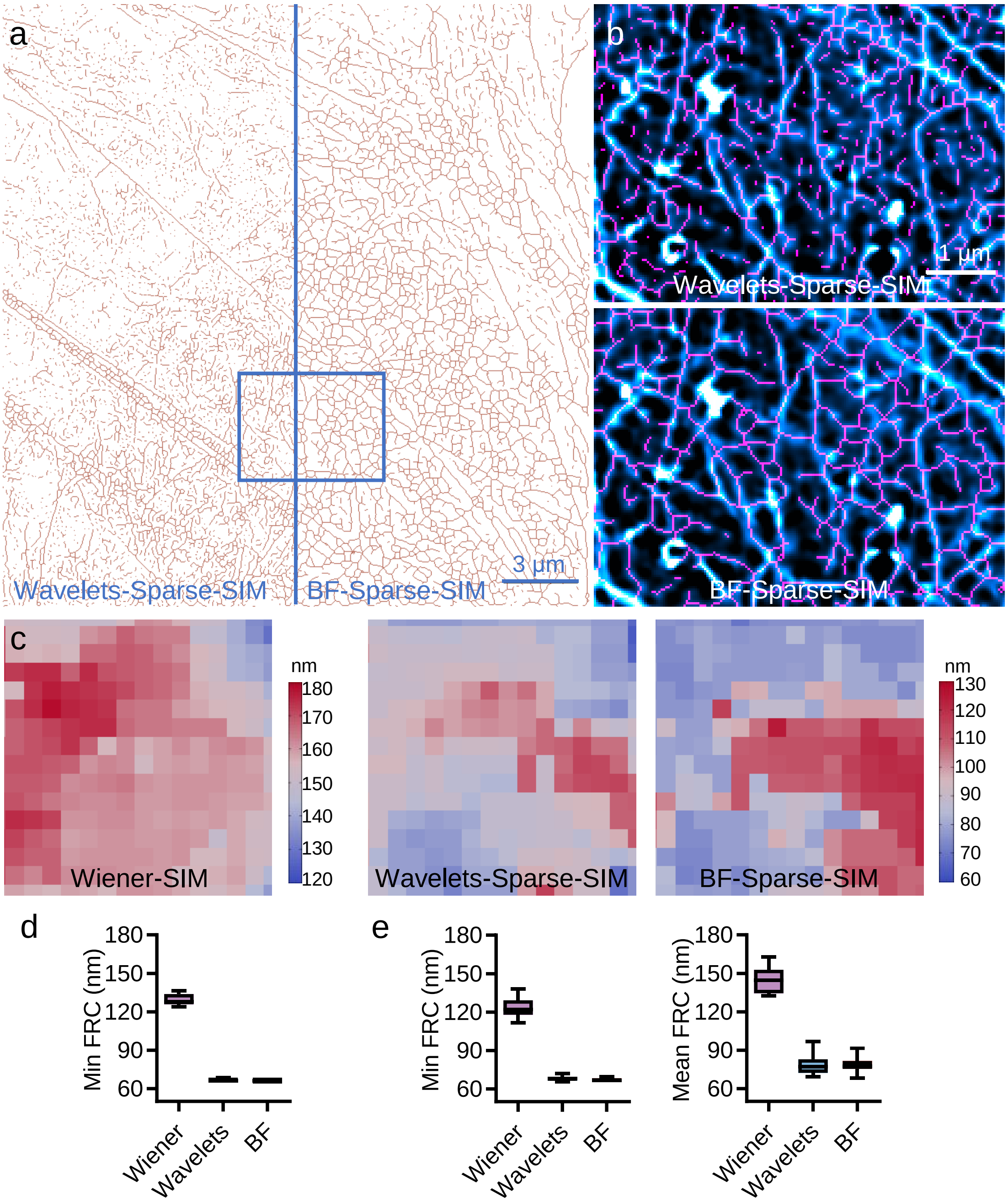

Supplementary Fig. 11 | Adding the BF step to Sparse-SIM increases the continuity of actin filaments. a, Skeletonized actin filaments under Sparse-SIM with and without the BF step in Fig. 2e. Both images were thresholded and skeletonized with the same parameters. **b,** The merged results of the SR image and the skeletonized actin of the enlarged ROIs in the blue box in **(a)**. **c,** FRC maps of images under the Wiener-SIM, Wavelets-Sparse-SIM, and BF-Sparse-SIM using PanelJ in Fig. 2e. **d**, The minimum FRC values (PanelJ) of SR images under the Wiener-SIM (129.5±1.2 nm), Wavelets-Sparse-SIM (66.8±0.3 nm), and BF-Sparse-SIM (66.4±0.1 nm), (n=3 biologically independent cells × 5 frames). **e**, The minimum and mean FRC values of images obtained under the Wiener-SIM, Wavelets-Sparse-SIM, and BF-Sparse-SIM n=3 biologically independent cells × 5 frames) calculated by the NanoJ-SQUIRREL.

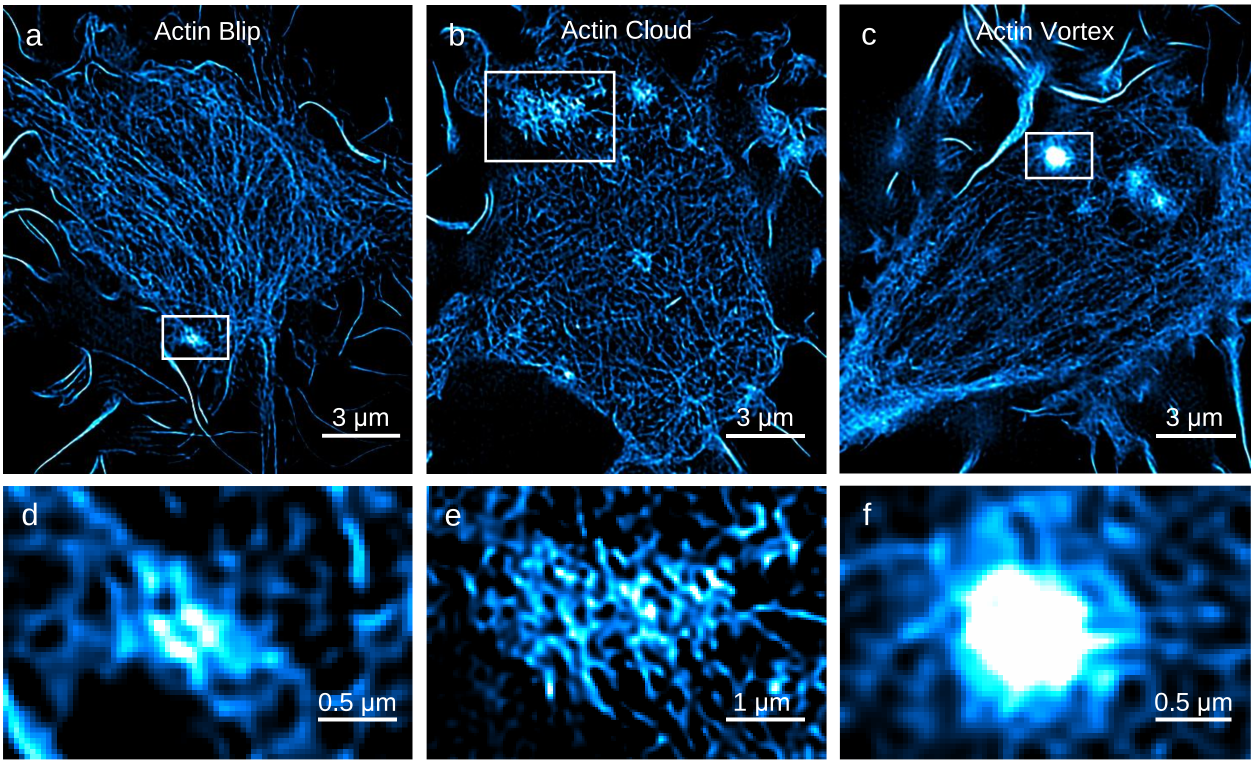

Supplementary Fig. 12 | Three types of transient actin dynamics. a,b,c, The representation examples of actin blip, actin cloud, and actin vortex in the living RAW264.7 cells. **d,e,f** Enlarged ROI in the white box in **(a-c)**.

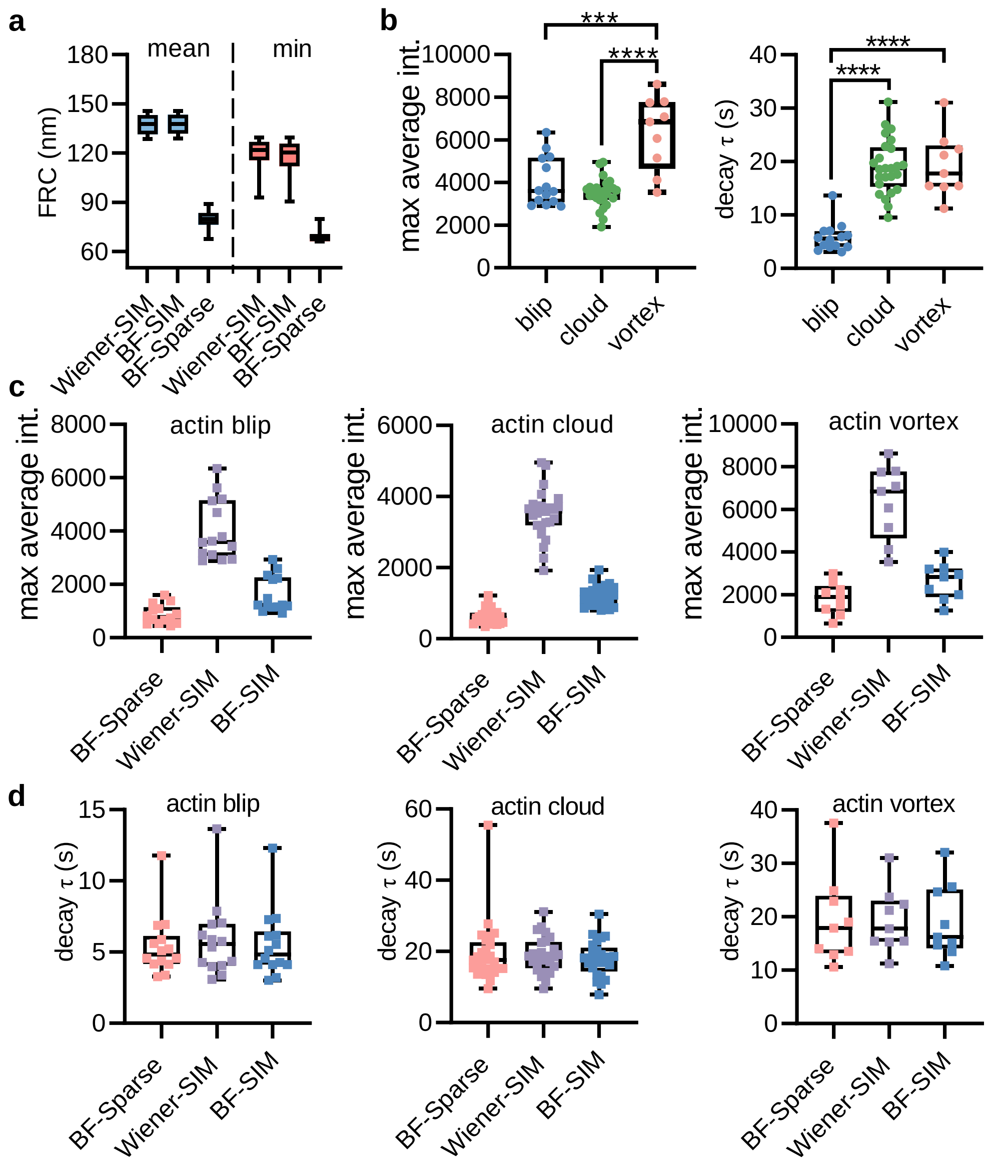

Supplementary Fig. 13 | Resolutions and actin event kinetics obtained by BF-Sparse-SIM, Wiener-SIM, and BF-SIM. a, The mean FRC resolutions of Wiener-SIM, BF-SIM, and BF-Sparse-SIM are 137.5±1.5 nm, 137.7±1.4 nm, and 79.3±1.5 nm, respectively. And the minimum FRC resolutions are 118.76±2.7 nm, 116±3.0 nm, and 69.2±0.9 nm , respectively (calculated by the NanoJ-SQUIRREL). **b**, The maximum average intensities and decay time constants of actin blips, clouds, and vortexes obtained by the Wiener-SIM. **c** and **d**, The maximum average intensities and decay time constants of actin blips, clouds, and vortexes from different reconstruction methods. All the statistics in this Figure are from the same 3 independent cells (n=3 cells) as in Figure 4, and the number of actin blip, cloud, and vortex events is 14, 25, and 9, respectively. We used the two-tailed unpaired Student's *t*-test for data in **(b)**, and no adjustments were made for multiple comparisons. *** $p < 0.001$, **** $p < 0.0001$. p=0.0010 (blip vs vortex) in the maximum average intensities.

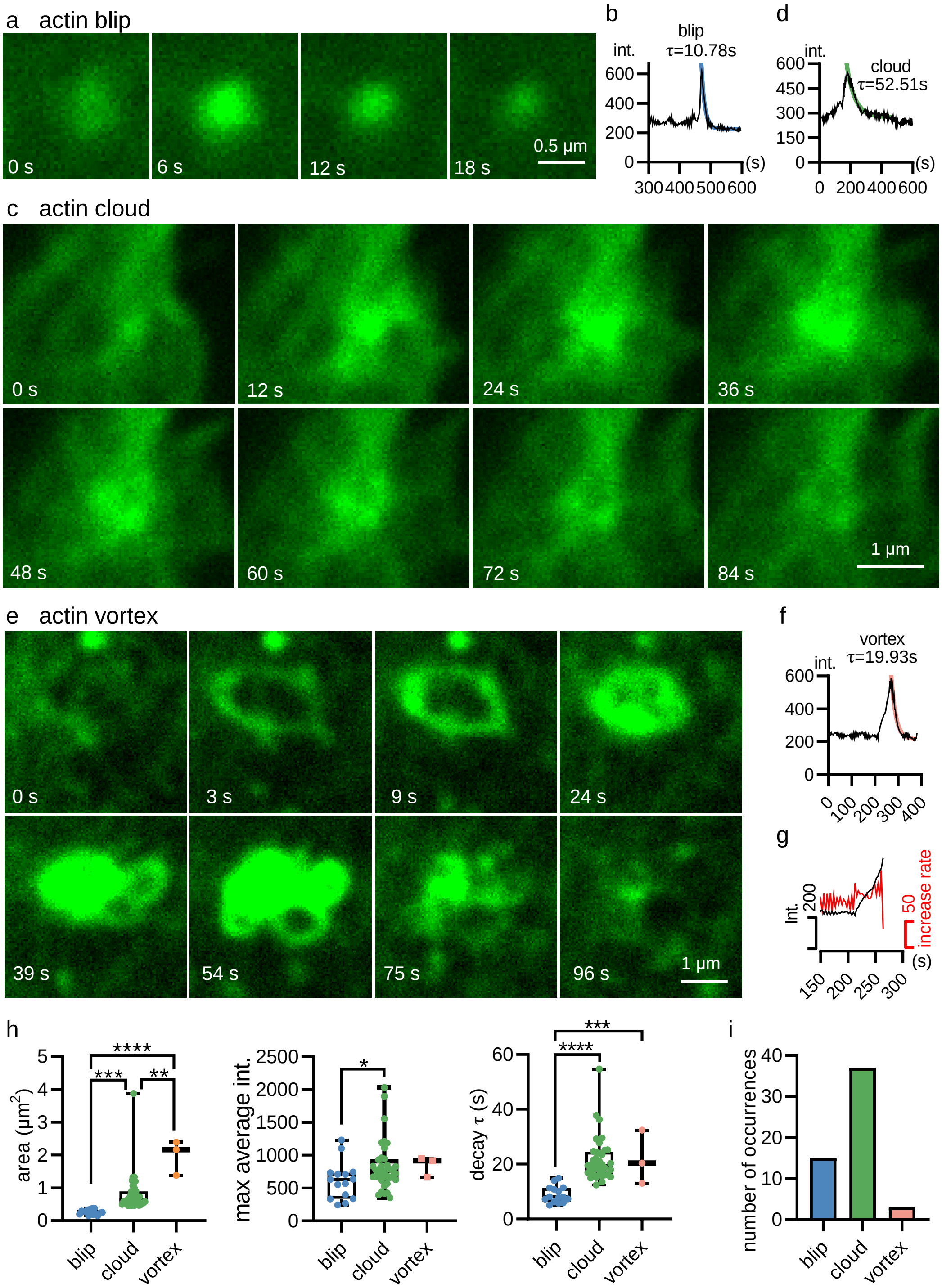

Supplementary Fig. 14 | Actin blips, clouds, and vortexes observed by the SD-SIM. **a**, **b**, A representative example of an actin blip event, which showed rapid increase and decay in fluorescence intensities. **c, d,** A representative example of an actin cloud event, demonstrating a slow increase, an intermediate plateau before the final slow decline in fluorescence intensities. **e-g,** A representative example of an actin vortex event that showed increased fluorescence intensities along with the spiral and outward-to-inward pattern **(e)**. It demonstrated an immediate increase and decay in fluorescence intensities **(f)**, and two speeds of intensity increases from the original intensity trace (black) and the differentiated intensity trace (red) **(g)**. **h,** The sizes, maximum average intensities, and decay time constants of these localized actin events. **i,** Occurrences of three localized actin events (blip 15, cloud 37, vortex 3, from 9 RAW264.7 cells). We used the two-tailed unpaired Student's t-test for data in **(h)**. $* \ p < 0.05$, $** \ p < 0.01$, $*** \ p < 0.001$, $**** \ p < 0.0001$. p=0.0006 (blip vs vortex), p=0.0018 (cloud vs vortex) in the area; p=0.0463 (blip vs cloud) in the maximum average intensities; p=0.0003 (blip vs vortex) in the decay time constants

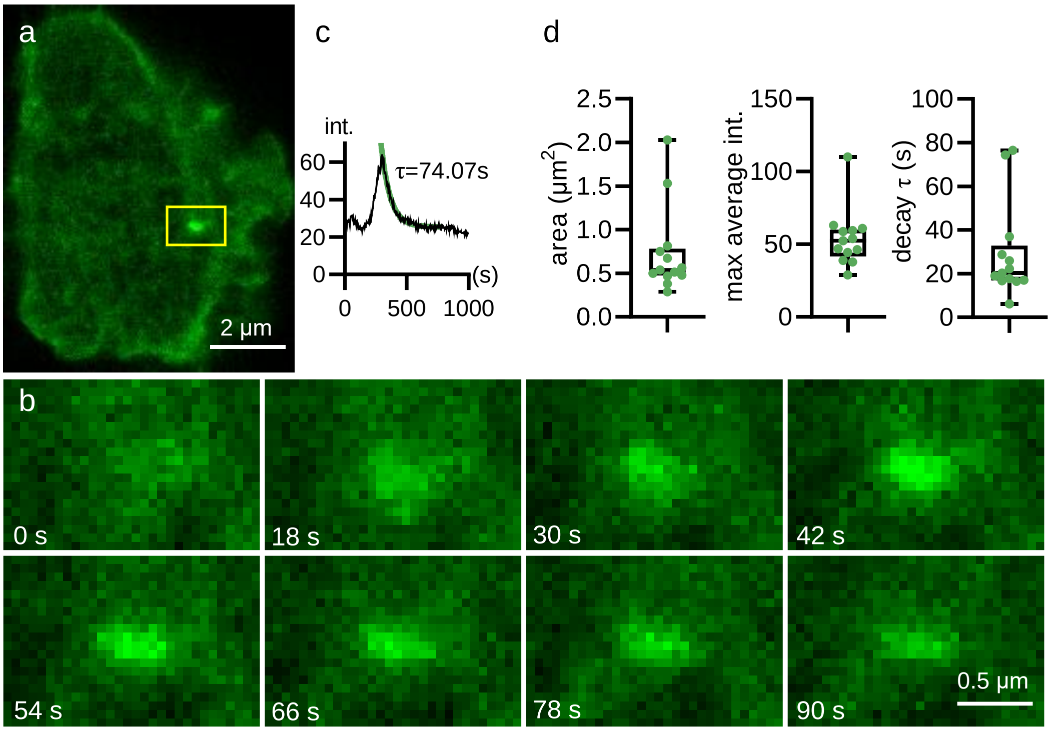

Supplementary Fig. 15 | Actin clouds observed by the STED. **a**,**b**, A representative example of actin cloud event observed by the STED, and different time points of the yellow boxed region in (**a**) were magnified and shown in (**b**). **c**, The rise and the decay of fluorescence intensities of the event shown in (**b**). **d,** The sizes (left, 0.733±0.137 μm^2), maximum average intensities (middle, 54.0±5.5), and decay time constants (right, 29.06±6.05 s) of all 13 events in 5 cells.

Supplementary Table.1 Actin dynamics revealed by BF-Sparse-SIM, Wiener-SIM, and BF-SIM.

Types	Area (μm^2)			Max average intensity			Decay τ (s)		
Method	BF-Sparse-SIM	Wiener-SIM	BF-SIM	BF-Sparse-SIM	Wiener-SIM	BF-SIM	BF-Sparse-SIM	Wiener-SIM	BF-SIM
blip	0.270	-	-	874.0	4031.5	1628.1	5.16	5.85	5.52
cloud	1.48	-	-	615.9	3499.5	1212.2	18.25	19.01	17.94
vortex	0.715	-	-	1814.1	6328.7	2613.1	18.50	19.27	19.02

Supplementary Table.2 Actin dynamics revealed by BF-Sparse-SIM, SD-SIM, and STED.

Types	Area (μm^2)			Max average intensity			Decay τ (s)		
Method	BF-Sparse-SIM	SD-SIM	STED	BF-Sparse-SIM	SD-SIM	STED	BF-Sparse-SIM	SD-SIM	STED
blip	0.270	0.262	-	874.0	612.3	-	5.16	8.93	-
cloud	1.48	0.817	0.733	615.9	831.2	54.0	18.25	21.71	29.06
vortex	0.715	1.978	-	1814.1	845.3	-	18.50	21.94	-

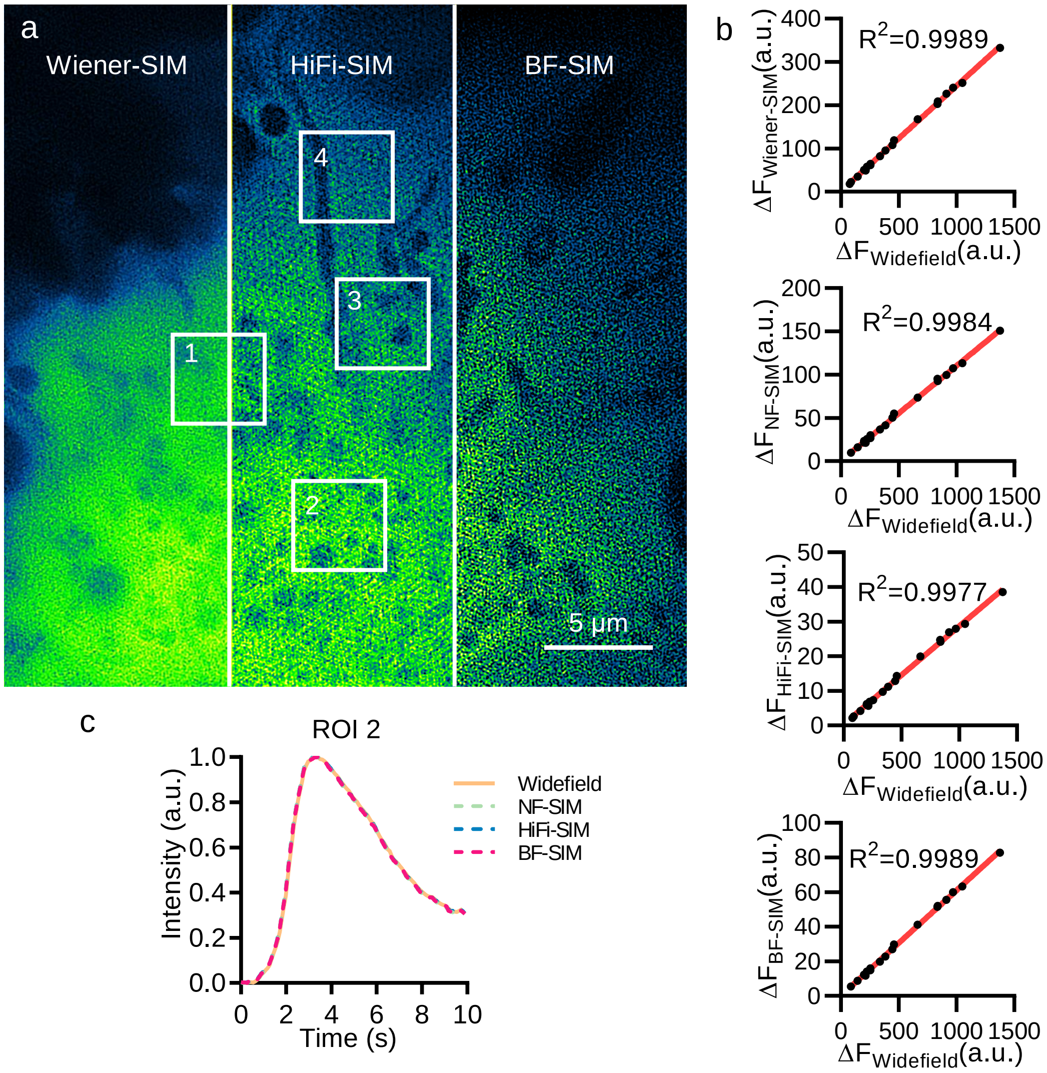

Supplementary Fig. 16 | Ca^{2+} transients under the BF-SIM are highly correlated to those under the widefield microscopy. a, A representative living COS-7 cell was transfected with GCaMP6s, stimulated with ATP. **b**, Increases in GCaMP6s fluorescence intensity from different macrodomains (5 cell × 4 regions) under the widefield exhibited linear relationships with those obtained under the Wiener-SIM, NF-SIM (attStrength=0.995), HiFi-SIM, and BF-SIM. **c**, ATP stimulated calcium traces from corresponding macrodomains in ROI 2 in (**a**).

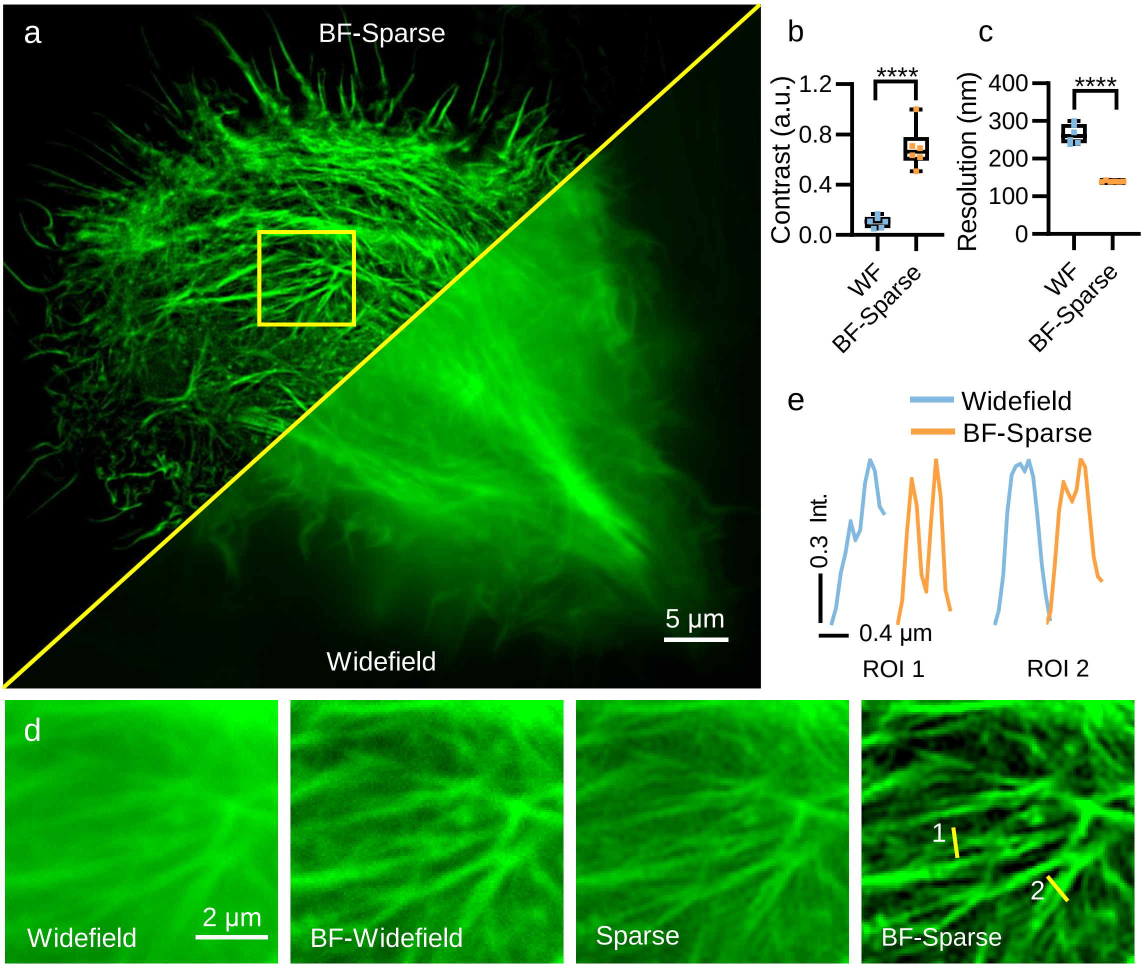

Supplementary Fig. 17 | BF-Sparse improves the contrast and resolution of widefield images. a, Actin filaments (LifeAct-EGFP) of a live U2OS cell seen under the widefield microscopy without (bottom right) or with (upper-left) BF-Sparse preprocessing. **b,** Normalized contrast ratios of WF images of actin filaments without (blue) and with (orange) BF preprocessing (n=6 biologically independent cells). **c,** FRC resolutions of WF images (264.93±10.49 nm, n=6), and WF with BF-Sparse (139.71±0.66 nm, n=6 biologically independent cells). **d,** Enlarged ROIs in **(a)** showing regions with WF, WF with BF, WF with Sparse, and WF with BF-Sparse preprocessing. **e,** Normalized intensity profiles along the yellow line in **(d)**. We used the two-tailed unpaired Student's *t*-test for the data in **(b,c)**. **** $p < 0.0001$.

References

1. Schneider, C. A., Rasband, W. S. & Eliceiri, K. W. NIH Image to ImageJ: 25 years of image analysis. *Nat. Methods* **9**, 671–675 (2012).
2. Sandison, D. R. & Webb, W. W. Background rejection and signal-to-noise optimization in confocal and alternative fluorescence microscopes. *Appl. Opt.* **33**, 603–615 (1994).
3. Rayleigh, Lord. V. *Investigations in optics, with special reference to the spectroscope. Lond. Edinb. Dublin Philos. Mag. J. Sci.* **9**, 40–55 (1880).
4. Huang, X. *et al.* Fast, long-term, super-resolution imaging with Hessian structured illumination microscopy. *Nat. Biotechnol.* **36**, 451–459 (2018).
