# Supplementary material for: Quantitative structured illumination microscopy via a physical model-based background filtering algorithm reveals actin dynamics
Source: Nat Commun. 2023 May 29;14:3089. doi: 10.1038/s41467-023-38808-8 (PMC10227022; doi:10.1038/s41467-023-38808-8)
Supplement: Supplementary file 3 — Description of Additional Supplementary Files [file 41467_2023_38808_MOESM3_ESM.docx]

**Description of Additional Supplementary Files**

Supplementary Movie 1

Description: Part I displays a widefield image of a COS-7 cell, which has been labeled with LifeActEGFP and reconstructed via traditional Wiener-SIM, HiFi-SIM, and BF-SIM methods (refer to Fig. 1b). In Part II, the performances of Wiener-SIM, HiFi-SIM, and BF-SIM are compared with each other on corresponding magnified regions of interest.

Supplementary Movie 2

Description: HiFi-SIM and BF-SIM have reconstructed the SR images of microtubules that were imaged layer by layer using multilayer two-beam interference SIM. The corresponding magnified regions of interest have also been included (refer to Supplementary Fig. 8), where the microtubules were shown in 3.0 μm size with an axial interval of 100 nm. The depth of the microtubules has been represented using pseudo-color.

Supplementary Movie 3

Description: In part I, widefield, fairSIM without or with BF preprocessing of mitochondria, which from COS-7 cells labeled with MitoTracker Green (cf., Supplementary Fig. 9). Part II provides a comparison of the widefield method, fairSIM without BF preprocessing, and fairSIM with BF preprocessing on the corresponding magnified regions of interest.

Supplementary Movie 4

Description: In Part I, a movie has been created showcasing the mitochondria reconstructed by four different methods - wiener-SIM, BF-SIM, Sparse without background suppression, and BF-Sparse-SIM. These images were obtained from COS-7 cells that were labeled with MitoTracker Green (refer to Supplementary Fig. 10a). Part II focuses on the magnified regions of interest of these four different methods, both in signal dense area and sparse area.

Supplementary Movie 5

Description: The movie of part I is endoplasmic reticulum reconstructed by wiener-SIM, BF-SIM, Sparse without background suppression, and BFSparse-SIM, respectively (cf., Supplementary Fig. 10b, from COS-7 cells labeled with mCherry-Cytb5ER). Part II provides a comparison of the magnified regions of interest of these four different methods, both in signal dense area and sparse area.

Supplementary Movie 6

Description: In Part I, the SR images of actin filaments (labeled with LifeAct-EGFP) in a living U2OS cell were reconstructed using two different methods - Waveltes-Sparse-SIM and BFSparse-SIM (refer to Fig. 2e). Part II provides a comparison between the performances of Waveltes-Sparse-SIM and BF-Sparse-SIM, focusing on the magnified regions of interest in the corresponding images.

Supplementary Movie 7

Description: The representation examples of local fast dynamics of actin blip, actin cloud, and actin vortex in living RAW264.7 cells (cf., Fig. 2 and Supplementary Fig. 12).
